# Negative association between higher maternal pre-pregnancy body mass index and breastfeeding outcomes is not mediated by DNA methylation

**DOI:** 10.1038/s41598-024-65605-0

**Published:** 2024-06-25

**Authors:** Hannah R. Elliott, Chloe L. Bennett, Doretta Caramaschi, Sinead English

**Affiliations:** 1 MRC Integrative Epidemiology Unit, University of Bristol, Bristol, UK; 2 Population Health Sciences, Bristol Medical School, University of Bristol, Bristol, UK; 3 School of Biological Sciences, University of Bristol, Bristol, UK; 4 Faculty of Health and Life Sciences, Department of Psychology, University of Exeter, Exeter, UK

**Keywords:** Breastfeeding, Maternal pre-pregnancy BMI, ALSPAC, DNA methylation, Epigenetic, Epigenome-wide association studies (EWAS), Epigenetics, Medical research, Molecular medicine

## Abstract

The benefits of breastfeeding for the health and wellbeing of both infants and mothers are well documented, yet global breastfeeding rates are low. One factor associated with low breast feeding is maternal body mass index (BMI), which is used as a measure of obesity. The negative relationship between maternal obesity and breastfeeding is likely caused by a variety of social, psychological, and physiological factors. Maternal obesity may also have a direct biological association with breastfeeding through changes in maternal DNA methylation. Here, we investigate this potential biological association using data from a UK-based cohort study, the Avon Longitudinal Study of Parents and Children (ALSPAC). We find that pre-pregnancy body mass index (BMI) is associated with lower initiation to breastfeed and shorter breastfeeding duration. We conduct epigenome-wide association studies (EWAS) of pre-pregnancy BMI and breastfeeding outcomes, and run candidate-gene analysis of methylation sites associated with BMI identified via previous meta-EWAS. We find that DNA methylation at cg11453712, annotated to PHTP1, is associated with pre-pregnancy BMI. From our results, neither this association nor those at candidate-gene sites are likely to mediate the link between pre-pregnancy BMI and breastfeeding.

## Introduction

Breastfeeding is associated with a multitude of benefits to mothers and infants, both in the short and long term, including reduced risk of breast cancer, diabetes and obesity, and improved cognition^1,2^. The World Health Organisation recommends exclusive breastfeeding (EBF) as the main infant feeding method for the first 6 months of life^3^. Despite health benefits, rates of breastfeeding initiation and duration remain low globally. A 2010 study on breastfeeding rates in the UK showed that, while 83% of mothers initiate breastfeeding, only 24% were EBF at 6 weeks, and 1% at 6 months^4^. These trends are apparent in countries with both high and low income, with evidence suggesting that increasing breastfeeding rates worldwide could lead to prevention of 823,000 deaths of children under 5 years old annually^2^. Strategies are required to increase breastfeeding rates, but for these to be most effective, understanding the mechanisms underlying reduced breastfeeding rates is essential.

Of several factors associated with reduced breastfeeding, maternal obesity is of particular concern. The prevalence of overweight and obesity among women of reproductive age is increasing globally: in 2016, it was estimated that 40% of women aged 18 or over were overweight^5,6^. Body mass index (BMI) denotes the weight of an individual relative to the square of their height and is used to categorise overweight and obesity^5,6^. Extensive studies have shown that obesity or high pre-pregnancy BMI are associated with lower breastfeeding initiation and shorter breastfeeding duration^6–9^. Potential causes behind this relationship include socio-cultural factors such as low self-esteem and body confidence, physical factors such as difficulties for the infant when latching to the breast, and physiological factors such as hormone imbalances or low milk supply in women who are overweight or obese^10–12^. Indeed, there is evidence that the second stage of lactogenesis (i.e., from 3 days up to one week after birth) is delayed in overweight women^13,14^. While the link between BMI and psychosocial factors associated with breastfeeding has been well explored^12,15^, there has been less investigation into the underlying biological or physiological mechanisms (but see^16,17^).

Here, we explore the biological association between high maternal pre-pregnancy BMI and reduced breastfeeding, focussing on the potential role of DNA methylation. DNA methylation is typically measured as attachment of a methyl group to a cytosine adjacent to a guanine nucleotide (referred to as a CpG site). It is a biological mechanism involved in regulation of gene expression that is influenced by both genetics and environment^18,19^. There is growing evidence from several cohort studies that there are epigenetic differences associated with increased BMI^20–31^, with loci in biological pathways involved in lipid metabolism, adipose tissue hypoxia, and inflammation, although the relevant loci are not always consistent across studies. For most sites identified, causal inference analyses indicate that the likely pattern is that increased BMI leads to changes in methylation rather than vice versa^23,25,28^. Breastfeeding has been associated with differential methylation profiles in offspring^32,33^. Studies have also addressed how the DNA methylation profile of mothers changes across pregnancy^34^, with differential methylation in loci associated with metabolism and mammary gland development. Less understood, however, is how maternal DNA methylation is associated with breastfeeding outcome, and how this might depend on pre-pregnancy BMI. For example, the physiological effect of delayed lactogenesis among overweight mothers may have an epigenetic association.

Here, we analyse data from a UK population study, the Avon Longitudinal Study of Parents and Children (ALSPAC), to investigate, first, the extent to which pre-pregnancy BMI is associated with breastfeeding outcomes, and second, whether any such associations are potentially mediated by BMI-associated methylation in pregnant mothers. To achieve the latter, we conducted epigenome-wide association studies (EWAS) of methylation and its association with pre-pregnancy BMI, breastfeeding initiation, and breastfeeding duration.

We also conducted specific candidate-gene analysis using a predetermined set of CpG sites known to be associated with BMI in adults, identified using a large meta-analysis of 9 cohorts (n = 17,034)^31^, to ascertain whether these sites are also associated with pre-pregnancy BMI in women and whether methylation at these sites is associated with breastfeeding practices. Any associations found between the traits of interest and DNA methylation within the maternal epigenome could be used to support the hypothesis that DNA methylation acts as a mediator in the relationship between increased pre-pregnancy BMI and reduced breastfeeding rates.

## Results

### Sample characteristics

The descriptive statistics generated for the ALSPAC sample used in our study, and the ARIES subset of ALSPAC in which DNA methylation data were collected are shown in Table 1. As explained in the Methods section, we measured breastfeeding outcome in terms of whether breastfeeding was initiated at all or not (breastfeeding initiation), and breastfeeding duration in months. ARIES mothers are broadly similar to the ALSPAC sample, in line with previous evidence^35^. Even though the comparisons are generally consistent, ARIES mothers have a lower prevalence of smoking during pregnancy; and a higher proportion of ARIES mothers initiated and maintained breastfeeding practice compared to mothers in the broader ALSPAC dataset. We only included mothers with non-missing information in the EWAS. As such, 724 samples were included in the EWAS of pre-pregnancy BMI, 718 samples were included in the EWAS of breastfeeding initiation and 602 individuals were included in the EWAS of breastfeeding duration. Descriptive statistics for individuals included in each EWAS are shown in Table 1.

**Table 1:** Comparison of the baseline characteristics for the exposure variables and covariates used in the EWAS between mothers included in ALSPAC only and the ARIES subset.

Variable	ALSPAC dataset	ARIES subset (n = 933)	ARIES subsets (EWAS)		
			BMI (n = 724)	Breastfeeding initiation (n = 718)	Breastfeeding duration (n = 602)
	Mean (standard deviation) OR %	Mean (standard deviation) OR %	Mean (standard deviation) OR %	Mean (standard deviation) OR %	Mean (standard deviation) OR %
Pre-pregnancy BMI (continuous)	n = 11,376	n = 861	n = 724	n = 718	n = 602
	22.9 (3.9)	22.8 (3.6)	22.7 (3.5)	22.7 (3.4)	22.5 (3.2)
Pre-pregnancy BMI (categorical)	n = 11,376	n = 861	n = 724	n = 718	n = 602
< 25	79.33%	82.69%	83.70%	83.98%	85.05%
25–29	15.10%	12.78%	12.15%	12.12%	11.46%
≥ 30	5.56%	4.53%	4.14%	3.90%	3.49%
Parity	n = 12,774	n = 902	n = 724	n = 718	n = 602
Nulliparous	44.97%	46.90%	49.17%	49.03%	49.50%
Multiparous	55.03%	53.10%	50.83	50.97%	50.50%
Age at delivery (years)	n = 11,539	n = 898	n = 724	n = 718	n = 602
	28.3 (4.8)	29.6 (4.3)	29.9 (4.2)	29.9 (4.2)	30.1 (4.1)
Smoked during pregnancy	n = 12,912	n = 916	n = 724	n = 718	n = 602
Yes	18.79%	9.28%	7.46%	7.38%	7.14%
No	81.21%	90.72%	92.54%	92.62%	92.90%
Education level	n = 11,413	n = 894	n = 724	n = 718	n = 602
Below A-Level	63.36%	48.55%	46.41%	45.96%	41.03%
A Level and above	37.64%	51.45%	53.59%	54.04%	58.97%
Occupation	n = 9855	n = 820	n = 724	n = 718	n = 602
Manual	19.90%	13.29%	12.57%	12.34%	11.13%
Non-manual	80.10%	86.71%	87.43%	87.60%	88.87%
Breastfeeding intention	n = 11,694	n = 890	n = 724	n = 718	n = 602
No	19.68%	10.22%	8.43%	8.36%	11.63%*
Maybe	14.19%	12.81%	12.71%	12.81%	
Yes	66.13%	76.97%	78.87%	78.83%	88.37%
Breastfeeding initiation (ever breastfed)	n = 12,035	n = 912	n = 724	n = 718	n = 602
Yes	77.25%	89.58%	90.11%	90.11%	100.00%
No	22.75%	10.42%	9.89%	9.90%	0.00%
Breastfeeding duration (categorised)	n = 10,875	n = 887	n = 724	n = 718	n = 602
Never	24.51%	13.19%	12.61%	12.57%	30.00%*
Up to 3 months	31.99%	28.18%	27.62%	27.71%	
Up to 5 months	12.83%	16.35%	16.29%	16.29%	18.61%
6 or more months	30.67%	42.28%	43.48%	43.43%	51.10%
Breastfeeding duration (months)	n = 7862	n = 754	n = 724	n = 718	n = 602
	5.7 (4.5)	6.4 (4.4)	6.5 (4.4)	6.5 (4.4)	6.5 (4.4)

*Counts of 5 individuals or fewer cannot be reported from the ALSPAC cohort. These categories were collapsed.

### Association between pre-pregnancy BMI and breastfeeding (initiation and duration)

In both univariate and multivariate models (including potentially confounding variables of breastfeeding intention, maternal smoking, age, occupation, parity, and education), increased pre-pregnancy BMI was associated with a lower likelihood of initiating breastfeeding (univariate model, n = 10,548; odds ratio OR [95% confidence interval CI] 0.96 [0.95, 0.97], z = − 6.62, p = 3.7 × 10^–11^; multivariate model, n = 7,70; OR [CI] 0.94 [0.92, 0.97], z = − 4.43, p = 9.41 × 10^–6^). Similarly, among mothers who did breastfeed, the duration of breastfeeding was lower for those mothers with higher pre-pregnancy BMI (effect of pre-pregnancy BMI [kg/m^2^] on breastfeeding duration (months): univariate model, n = 7166; Beta [CI] − 0.11 [− 0.14, − 0.08], t = − 7.26, p = 4.38 × 10^–13^; multivariate model, n = 5645; Beta [CI] − 0.09 [− 0.12, − 0.06], t = -5.64, p = 1.83 × 10^–8^). When we analysed breastfeeding duration as a categorical variable, we also find that higher pre-pregnancy BMI (kg/m^2^) is associated with shorter breastfeeding outcomes (univariate model, n = 9761; OR [CI] 0.95 [0.94, 0.95], t = − 11.52, p = 9.92 × 10^–31^; multivariate model, n = 7298; OR [CI] 0.94 [0.93, 0.96], t = − 8.58, p = 9.43 × 10^–18^). For results describing all covariates, see Tables S1–S3 (ESM). Note that we found qualitatively similar results when we considered categorised pre-pregnancy BMI (rather than continuous) as a covariate (Tables S1–S3) and when we ran models without breastfeeding intention as a covariate (Tables S1–S3), given that pre-pregnancy BMI may be associated with intention to breastfeed. We also found similar results when we analysed breastfeeding duration (in months) as a time-to-event process, with proportional hazards model and considering pre-pregnancy BMI as a categorical variable (Table S4, Fig. S1, ESM).

### Epigenome-wide association analyses

#### Pre-pregnancy BMI

**Figure 1:**
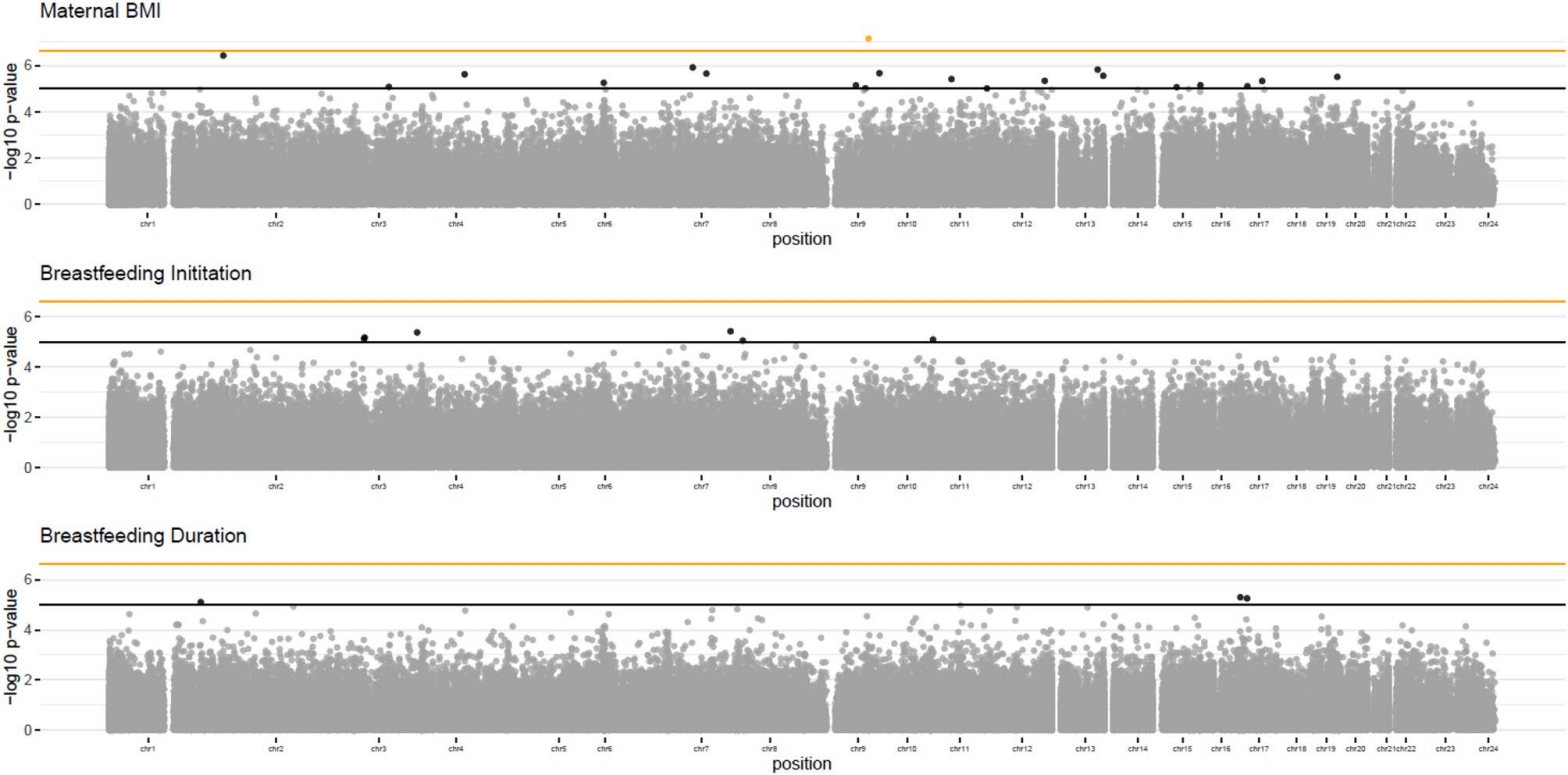
Manhattan plots showing the association between DNA methylation and phenotype (pre-pregnancy BMI, breastfeeding initiation, and breastfeeding duration) measured in maternal blood samples from the ALSPAC cohort. Each point on the plots represents an individual CpG site. The orange threshold line represents a genome wide threshold of p < 2.4 × 10^–7^ and CpG sites with p-values below this threshold are highlighted in orange. The black threshold line represents a relaxed threshold of p < 1.0 × 10^–5^ and CpG sites with p-values below this threshold are highlighted in black.

In multivariate analysis (including potentially confounding variables of breastfeeding intention, maternal smoking, age, occupation, parity, and education, cell composition estimates and 10 surrogate variables), one CpG site was identified below the genome wide threshold (*p* < 2.4 × 10^–7^) when pre-pregnancy BMI was modelled as the exposure variable (see Fig. 1 and Table 2). A total of 20 CpG sites were associated with pre-pregnancy BMI at *p* < 1.0 × 10^–5^ in the multivariate analysis (Table 2). The lambda value (λ = 1.29) indicated minor inflation of the results. We used bacon^36^ to correct residual inflation in this EWAS, but found this adjustment did not alter CpG sites identified at the specified *p*-value thresholds and did not remove the residual inflation observed (λ = 1.19). All test statistics related to EWAS of pre-pregnancy BMI are shown in Table S5, ESM.

**Table 2:** The associations between DNA methylation and maternal phenotype for fully adjusted EWAS at p < 1.0 × 10^–5^ across the three EWAS conducted in ALSPAC.

probeID	Gene symbol	Chr	Position	Pre-pregnancy BMI			Breastfeeding initiation			Breastfeeding duration		
				coef	SE	*p-*value	coef	SE	*p-*value	coef	SE	*p-*value
cg11453712	PHPT1	**9**	**139,741,803**	**1.5E−03**	**2.8E−04**	**6.9E−08**	− 2.1E+00	8.9E+00	8.1E−01	2.0E+00	6.7E+00	7.7E−01
cg05698294		**2**	**3,063,249**	**3.9E−03**	**7.6E−04**	**3.7E−07**	9.4E−01	3.4E+00	7.8E−01	1.9E+00	2.5E+00	4.6E−01
cg00831126		**7**	**56,605,325**	**3.6E−03**	**7.3E−04**	**1.2E−06**	− 3.1E+00	4.2E+00	4.6E−01	3.2E−01	2.5E+00	9.0E−01
cg00224563	PCCA	**13**	**100,741,287**	**−9.8E−05**	**2.0E−05**	**1.5E−06**	− 1.2E+02	1.3E+02	3.7E−01	1.2E+02	9.5E+01	2.2E−01
cg26176206		**10**	**22,518,170**	**− 8.5E−05**	**1.8E−05**	**2.2E−06**	− 4.3E+01	1.5E+02	7.8E−01	2.2E+01	1.1E+02	8.4E−01
cg11683732	C7orf23	**7**	**86,849,468**	**− 1.7E−04**	**3.6E−05**	**2.2E−06**	1.4E+02	8.0E+01	8.8E−02	5.6E+01	5.3E+01	2.9E−01
cg26612230	PDLIM5	**4**	**95,508,526**	**− 7.0E−04**	**1.5E−04**	**2.4E−06**	3.1E+01	1.9E+01	1.1E−01	− 2.2E+01	1.4E+01	1.1E−01
cg02188190		**13**	**112,894,616**	**− 3.5E−03**	**7.5E−04**	**2.8E−06**	4.4E+00	3.3E+00	1.8E−01	− 8.9E−01	2.5E+00	7.2E−01
cg08496404	ISOC2	**19**	**55,969,333**	**6.3E−04**	**1.3E−04**	**3.1E−06**	1.9E+01	2.2E+01	3.7E−01	9.2E+00	1.4E+01	5.2E−01
cg20774552		**11**	**46,415,316**	**− 1.9E−03**	**4.0E−04**	**3.9E−06**	− 1.0E−01	7.3E+00	9.9E−01	3.6E+00	4.7E+00	4.5E−01
cg03869225	TESC	**12**	**117,477,105**	**− 1.5E−03**	**3.3E−04**	**4.6E−06**	1.7E+01	7.8E+00	3.0E−02	3.0E+00	5.8E+00	6.1E−01
cg07168556	WFIKKN2	**17**	**48,912,483**	**3.2E−03**	**7.0E−04**	**4.7E−06**	− 2.2E+00	4.4E+00	6.2E−01	1.5E+00	2.6E+00	5.8E−01
cg18086243		**6**	**30,924,004**	**− 3.2E−03**	**7.0E−04**	**5.6E−06**	− 6.5E+00	4.4E+00	1.4E−01	2.0E+00	2.7E+00	4.5E−01
cg04484916	SRRM2	**16**	**2,801,730**	**− 2.4E−03**	**5.4E−04**	**7.1E−06**	3.4E+00	4.7E+00	4.7E−01	− 1.0E+00	3.5E+00	7.7E−01
cg13546538	CTNNAL1	**9**	**111,754,815**	**− 1.2E−03**	**2.7E−04**	**7.2E−06**	− 3.0E−01	1.0E+01	9.8E−01	4.5E−01	7.0E+00	9.5E−01
cg01590416	ZNF287	**17**	**16,472,754**	**− 1.4E−04**	**3.0E−05**	**7.9E−06**	2.5E+01	8.5E+01	7.7E−01	1.0E+01	6.5E+01	8.8E−01
cg02749148	KLF15	**3**	**126,076,844**	**7.4E−04**	**1.6E−04**	**8.4E−06**	2.0E+01	1.7E+01	2.3E−01	− 1.4E+01	1.1E+01	2.2E−01
cg17447014	MYO5A	**15**	**52,821,424**	**− 8.1E−05**	**1.8E−05**	**8.6E−06**	2.4E+01	1.5E+02	8.7E−01	1.5E+02	1.0E+02	1.4E−01
cg13503179	FNBP1	**9**	**132,695,505**	**2.3E−03**	**5.2E−04**	**9.7E−06**	− 2.9E+00	5.3E+00	5.8E−01	− 2.1E+00	3.7E+00	5.8E−01
cg01162104		**11**	**124,709,577**	**− 9.8E−05**	**2.2E−05**	**9.8E−06**	1.1E+02	1.3E+02	4.1E−01	1.3E+02	8.7E+01	1.3E−01
cg16311186	RAB19	7	140,114,137	− 2.7E−04	2.3E−04	2.4E−01	**7.1E+01**	**1.5E+01**	**3.7E−06**	− 3.6E+00	8.4E+00	6.7E−01
cg00902153	LPP	3	188,506,715	− 1.7E−04	2.5E−04	5.0E−01	**− 4.5E+01**	**9.8E+00**	**4.1E−06**	3.6E+00	7.7E+00	6.4E−01
cg08123528	RYBP	3	72,438,132	− 1.1E−03	6.2E−04	8.4E−02	**− 1.8E+01**	**4.1E+00**	**6.6E−06**	− 3.1E−01	3.0E+00	9.2E−01
cg09009983	FOXP1	3	71,203,748	3.7E−04	3.4E−04	2.7E−01	**− 3.4E+01**	**7.5E+00**	**7.4E−06**	5.2E+00	6.0E+00	3.9E−01
cg26217318	OR51I2	11	5,474,584	− 5.8E−05	3.4E−04	8.6E−01	**− 3.9E+01**	**8.8E+00**	**8.0E−06**	− 2.1E−01	5.9E+00	9.7E−01
cg23015041	PRAGMIN	8	8,176,714	5.1E−04	4.2E−04	2.2E−01	**2.8E+01**	**6.4E+00**	**8.8E−06**	− 2.0E+00	4.7E+00	6.7E−01
cg03986557	NXN	17	728,927	− 8.3E−05	2.4E−04	7.3E−01	− 4.6E+00	1.2E+01	6.9E−01	**− 3.8E+01**	**8.2E+00**	**5.0E−06**
cg08192143	ZNF286A	17	15,608,033	3.4E−04	3.7E−04	3.5E−01	7.1E+00	7.2E+00	3.3E−01	**− 2.3E+01**	**5.1E+00**	**5.5E−06**
cg12718426	PPP1R12B	1	202,430,474	2.3E−04	4.6E−04	6.1E−01	7.6E+00	6.5E+00	2.5E−01	**1.8E+01**	**4.1E+00**	**7.8E−06**

*Coef* coefficient from multivariate model (intention to breastfeed, maternal smoking, age, occupational social class, parity, and education in all models; additional adjustment of pre-pregnancy BMI in breastfeeding models), *SE* standard error from multivariate model, *P-value* p-value from multivariate model, *Gene symbol* mapped gene based on Illumina mapping, *Chr* chromosome, *Position* base pair position of CpG. Annotations are based on hg19, build 37.

Significant values are given in bold.

We ran a differentially methylated region (DMR) analysis to test if any individual CpG sites identified in the EWAS of pre-pregnancy BMI were part of a larger associated region. We identified 44 DMRs. Of these DMRs, we identified a 622 base-pair DMR on chromosome 7 where the representative CpG, cg11683732, had an EWAS *p* value of *p* < 1.0 × 10^–5^) (all DMRs are shown in Table S6, ESM).

#### Breastfeeding initiation

In the multivariate model (including breastfeeding intention, maternal smoking, age, occupation, parity, education and pre-pregnancy BMI, cell composition estimates and 10 surrogate variables), we did not identify any CpG sites associated with breastfeeding initiation below the genome wide threshold (*p* < 2.4 × 10^–7^, Fig. 1). Breastfeeding initiation was associated with 6 CpGs in the multivariate model at *p* < 1.0 × 10^–5^ (Fig. 1 and Table 2). The lambda value (λ = 1.27) indicates minor inflation of the results. We identified four DMRs, although all the representative CpGs had p-value of p > 1.0 × 10^–5^ (all DMRs are shown in Table S6, ESM).

We ran two further analyses. First, a multivariate model as above but excluding pre-pregnancy BMI as a covariate. Second, a multivariate model as above but including BMI as a categorical variable (defined according to WHO guidelines; see Methods). In the multivariate model not including BMI, we did not identify any CpG sites associated with breastfeeding initiation below the genome wide threshold (*p* < 2.4 × 10^–7^) and identified 4 CpGs at *p* < 1.0 × 10^–5^. A lambda value of 1.21 indicated minor inflation of *p-*values. In the multivariate model including BMI as a categorical variable, we did not identify any CpG sites associated with breastfeeding initiation below the genome wide threshold (*p* < 2.4 × 10^–7^) and identified 5 CpGs at *p* < 1.0 × 10^–5^. A lambda value of 1.24 indicated minor inflation of *p-*values. All test statistics related to EWAS of breastfeeding initiation are shown in Table S7, ESM.

#### Breastfeeding duration

In multivariate analysis (including breastfeeding intention, maternal smoking, age, occupation, parity, education and pre-pregnancy BMI, cell composition estimates and 10 surrogate variables), we did not identify any CpG sites associated with breastfeeding duration below the genome wide threshold (*p* < 2.4 × 10^–7^, Fig. 1). Continuously measured breastfeeding duration was associated with 3 CpG sites in the multivariate model at *p* < 1.0 × 10^–5^ (Fig. 1 and Table 2). The lambda value (λ = 1.02) showed no genome wide inflation of *p-*values versus those expected. We identified two DMRs, although all the representative CpGs had p-value of p > 1.0 × 10^–5^ (both DMRs shown in Table S6, ESM).

We ran two further analyses. First, a multivariate model as above but excluding BMI as a covariate. Second, a multivariate model as above but including BMI as a categorical variable. In the multivariate model not including BMI, we did not identify any CpG sites associated with breastfeeding initiation below the genome wide threshold (*p* < 2.4 × 10^–7^). Breastfeeding initiation was associated with 4 CpGs in the multivariate model at *p* < 1.0 × 10^–5^. The lambda value (λ = 1.02) indicated no inflation of *p*-values. In the multivariate model including BMI as a categorical variable, we did not identify any CpG sites associated with breastfeeding initiation below the genome wide threshold (*p* < 2.4 × 10^–7^) and identified 4 CpGs at *p* < 1.0 × 10^–5^. A lambda value of 1.03 indicated no inflation of *p-*values. All test statistics related to EWAS of breastfeeding duration are shown in Table S8, ESM.

### Candidate gene analysis

The results for the candidate gene analysis found that none of the 1236 CpG sites drawn from the Do et al. meta-EWAS analysis of BMI^31^ met the *p-*value threshold of 0.05/1236 = 4.05 × 10^–05^ (Table 2) in ALSPAC EWAS of BMI, breastfeeding initiation or breastfeeding duration. Do et al*.*^31^ comprises a discovery meta-EWAS of BMI including 17,034 individuals of European (n = 11,220), African (n = 3134) and South Asian (n = 2680) descent, with replication in the Women’s Health Initiative (n = 4822)^31^. ALSPAC test statistics for the CpG sites identified in Do et al*.* are shown in Table S9, ESM. We evaluated the consistency in direction of effects between our EWAS of pre-pregnancy BMI and results reported by Do et al*.* and calculated the proportion of CpGs which had the same direction of effect using a binomial test of the null hypothesis that the proportion is equal to 0.5. 921/1236 CpG sites showed consistency of direction of effect estimate, binomial test *p-*value = 3.22 × 10^–69^. We therefore demonstrate that there is weak evidence for association between BMI and DNA methylation in pregnant women in the ALSPAC cohort, but effect estimates are consistently in the same direction as previously reported in the literature. None of the CpG sites identified to be associated with BMI in the Do et al*.* study are, however, associated with the breastfeeding measures assessed in ALSPAC.

### Mediation analysis

We used the Sobel test to assess if the relationship between pre-pregnancy BMI and breastfeeding (initiation or duration) was mediated by methylation. We tested 29 CpGs (see Table 2), identified from the EWAS on pre-pregnancy BMI, breastfeeding initiation and breastfeeding duration at *p* < 1.0 × 10^–5^. We did not identify any evidence of mediation in this analysis. Results are shown in Table S10, ESM.

## Discussion

In this study, we first confirmed previously established associations between high maternal pre-pregnancy BMI and lower initiation and duration of breastfeeding, using analysis of the UK ALSPAC population study. We then conducted EWAS to identify whether maternal pre-pregnancy BMI and breastfeeding practice were associated with DNA methylation at CpG sites in the maternal genome, which could act as potential mediators in the relationship between increased pre-pregnancy BMI and lowered breastfeeding rates. We found BMI to be associated with DNA methylation at one CpG site and putatively associated with further 19 CpG sites. One of these sites, cg11683732, was part of a 622 base pair DMR. However, none of these sites were associated with breastfeeding initiation or duration. Moreover, we did not find any evidence that methylation of 29 CpGs (drawn from our EWAS on pre-pregnancy BMI, breastfeeding initiation and duration) mediates the relationship between pre-pregnancy BMI and breastfeeding outcome in our dataset.

We find a negative association between higher pre-pregnancy BMI and breastfeeding outcome—both in terms of whether it was initiated and how long it lasted—in line with several other studies on this topic across diverse cohorts^6,8,11^. Given that obesity rates are rising rapidly across the world, and breastfeeding rates remain low despite known benefits, studies investigating why high-BMI mothers are less likely to breastfeed their babies could help inform policy to improve breastfeeding outcome. We acknowledge limitations of using BMI as a measure of obesity^37^, yet appreciate that it is a practical measure and can be used at scale in population studies such as ALSPAC.

Potential factors explaining the replicated negative relationship between BMI and breastfeeding outcome include delayed lactogenesis. The second stage of lactogenesis, which occurs on average around 3 days up to a week after birth, is more likely to be delayed in overweight women^13,14^. We originally hypothesised that biological factors behind this phenomenon could be captured by epigenetic biomarkers in peripheral blood during pregnancy. Finding such biomarkers could have implications in identifying women in need of extra support and could further our understanding of the mechanisms behind delayed lactogenesis. However, we did not find strong evidence to support our hypothesis. Other potential factors that have been previously linked with reduced breastfeeding outcomes are parity, delivery method, experience of epidural, and infant behaviour after birth^13,14^. We adjusted our analyses for parity as a potential confounder in the associations between pre-pregnancy BMI and breastfeeding. Further studies should explore the other factors as potential mediators between high BMI and low breastfeeding initiation and duration as done by Martin et al*.*^17^, who find an association between pre-pregnancy BMI and gestational weight gain on breast feeding duration.

A robust relationship between BMI and DNA methylation was evidenced by previous large epigenome-wide association studies in the general population^25,30,31^, therefore we hypothesised that we would find a similar link in UK pregnant women. Although less powered, our study found associations in the same directions as found by the largest EWAS of BMI. We also found novel associations, though we are cautious about these unless replicated in other cohorts. The strongest association was with cg11453712, which is located on the PHPT1 gene. The PHPT1 gene is expressed in the mammary gland^38^ and encodes a phosphatase not previously linked to obesity or other health traits^39^. DNA methylation at cg11453712 has been previously linked to age^40^ and is associated with nearby genetic variants^41^. The CpG is located ~ 2 Kb upstream the transcription start site near regulatory elements (from a look-up on UCSC browser)^42^. To our knowledge, this is the first study investigating this question in pregnant women, and our results suggest that during pregnancy the relationship between pre-pregnancy BMI and DNA methylation might be altered, potentially due to immune-metabolic changes typical of pregnancy^43,44^. Previous studies that attempted to investigate the link between pre-pregnancy BMI and DNA methylation during pregnancy focussed on candidate genes encoding leptin and adiponectin and found associations^45^. Neither of these loci appear on our top list of genes associated with BMI, however, using the relaxed *p-*value threshold.

Our EWAS of breastfeeding outcome did not identify CpG sites whose methylation in the peripheral blood of pregnant women is associated with breastfeeding outcomes in our study. To our knowledge, this is the first study examining this link and we conducted an epigenome-wide scan across more than 450,000 CpG sites. It is possible that if there are true associations, their effect sizes will be too small to detect with our sample. The lack of study populations with DNA methylation data on pregnant women limited our sample size. However, if the top associations found with breastfeeding using the relaxed threshold are true, they suggest implications of the LPP and NXN genes. Methylation at these genes in the offspring at birth was also associated with pre-pregnancy BMI and pre-pregnancy overweight/obesity in a previous study, though at different CpG sites in the same loci^46^. However, DNA methylation at these genes was not associated with pre-pregnancy BMI in the antenatal mothers’s samples in our study. This suggests that even if there was an intergenerational effect from pre-pregnancy BMI to breastfeeding via DNA methylation, this would most likely happen via alterations of fetal development and with consequent challenges around delivery and in the baby’s feeding behaviour, rather than the mother’s own ability to lactate. In support of this possibility, a recent study shows that early pregnancy BMI is linked with DNA methylation in placental tissue^47^.

We also conducted a targeted approach, analysing candidate CpG sites previously found to be associated with BMI from a broader EWAS^31^. Similar to our EWAS analysis, we did not find support that methylation associated with BMI is linked with reduced breastfeeding outcomes. These results, together with the EWAS results and formal mediation analysis, do not support our hypothesis that DNA methylation is implicated in the link between pre-pregnancy BMI and breastfeeding practice. It is possible, however, that biological mechanisms unrelated to peripheral blood DNA methylation are involved in mediating the link between BMI and reduced breastfeeding. Further studies should be focused on elucidating these mechanisms. Another explanation is that biological mechanisms are less involved and that social and cultural aspects play a stronger role in the negative association between maternal overweight and breastfeeding outcome. For instance, many women may breastfeed their infants for a shorter period than initially planned because of discouraging factors such as employer support, husband involvement and social attitudes^48^. Moreover, studies have shown there can be a general negative public attitude towards breastfeeding^49^, and it is possible that overweight women are more vulnerable to these attitudes^50^. In any case, considering the challenges that overweight women face around breastfeeding, more dedicated support would be beneficial to increase breastfeeding rates.

Despite the strength of our study being that we addressed a novel question in a well-characterised, intergenerational population study, our study has several limitations. First, we had a small sample size for the methylation studies. Although the initial ALSPAC cohort recruited more than 14,000 pregnancies, the subsample with antenatal blood samples and data on pre-pregnancy BMI, breastfeeding outcome and covariate data is less than 1,000 mother–child pairs. This is considered an under-powered sample size for EWAS studies of outcomes such as BMI and it is increasingly common to combine multiple cohorts to greatly increase the number of subjects. For example, Sharp et al.^46^ were able to meta-analyse the association between maternal pre-pregnancy BMI and methylation in the peripheral blood of offspring across 19 longitudinal cohorts, resulting in a sample size of 9,340. Obtaining a sample size of this magnitude increases internal and external validity and reliability of results, as natural variation can greatly affect DNA methylation and hence the outcomes of the study^51^. Second, our analyses were not replicated. Future similar studies should be conducted to further investigate the association between increased pre-pregnancy BMI and DNA methylation in antenatal blood and how this methylation is associated with breastfeeding practice.Third, our EWAS was restricted to analysis of peripheral blood cells. The advantage of using peripheral blood as a surrogate tissue is that it can be collected in large quantities and is easy to store. In the context of this study, to assess the association between breastfeeding practice and changes to the DNA methylation profile, breast tissue samples (preferably prenatally or during the period where the mother breastfeeds) would be the optimal sample tissue. This would lead to more accurate and compelling analyses of associations between breastfeeding practice and breast tissue DNA methylation.

In conclusion, this study provides the first insight into the maternal epigenome and its association with maternal pre-pregnancy BMI and breastfeeding initiation and duration. The results from this study did not support our mediation hypothesis, that DNA methylation could explain the putative pattern of reduced breastfeeding among overweight mothers, yet owing to our underpowered study we are cautious to infer that BMI-associated methylation is not associated with reduced breastfeeding at all. Our novel investigation into the relationship between maternal exposures such as BMI and breastfeeding on maternal DNA methylation provides the framework for more in-depth study into the physiological mechanisms that impede a mother’s ability to breastfeed, and future studies could expand our analyses to more cohorts, and consider other biological pathways.

## Methods

### Study cohort

The Avon Longitudinal Study of Parents and Children (ALSPAC) is a birth cohort study that investigates modifiable influences on child health and development^52,53^. Pregnant women resident in Avon, UK, with expected dates of delivery between 1st April 1991 and 31st December 1992 were invited to take part in the study. The initial number of pregnancies enrolled was 14,541. Of the initial pregnancies, there was a total of 14,676 foetuses, resulting in 14,062 live births and 13,988 children who were alive at 1 year of age.

### Ethical approval

Ethical approval for the study was obtained from the ALSPAC Ethics and Law Committee and the Local Research Ethics Committees. Informed consent for the use of data collected via questionnaires and clinics was obtained from participants following the recommendations of the ALSPAC Ethics and Law Committee at the time. Consent for biological samples has been collected in accordance with the Human Tissue Act (2004).

### Study measures

#### Maternal pre-pregnancy body mass index (BMI)

BMI is the metric currently used for defining anthropometric height/weight characteristics and typically categorizing them into groups, and its common interpretation is that it is a measure of extent of overweight and obesity^5,6^. We used self-reported measures of pre-pregnancy weight (in kilograms) and height (in metres) from the ‘About Yourself’ mother-completed questionnaire which can be found in the ALSPAC data dictionary. Pre-pregnancy BMI was calculated as weight (kg)/height (m^2^) as a continuous variable; and analyses were repeated with pre-pregnancy BMI categorised as ˂ 25, 25–29 or ≥ 30, following standard WHO categorisation^54^.

#### Breastfeeding outcome

Data regarding breastfeeding outcome were collected from two questionnaires administered to the mother when the child was 6 months and 15 months of age respectively. Three different measures of breastfeeding were used: (1) breastfeeding initiation, a binary indicator (‘yes’ or ‘no’) of whether the mother had ever initiated any breastfeeding before the child had reached 6 months of age; and, for those mothers who initiated breastfeeding, (2) continuous breastfeeding duration, measured as the age in months (from 0 to 15) of the child when breastfeeding stopped, derived from the 15-month questionnaire, and (3) categorical breastfeeding duration, i.e. never, up to 3 months, 3–5 months and 6 or more months, derived from the 6-month questionnaire, as this provided data for a larger number of mothers. Note that these variables encompassed any measure of breastfeeding (i.e., combined both exclusive and mixed feeding methods). Further details on how these variables were derived can be found in the ‘My Son/My Daughter’ mother-completed questionnaire in the ALSPAC data dictionary.

#### DNA methylation

DNA methylation data was obtained using Illumina 450 k BeadChip arrays from the Accessible Resource for Integrated Epigenomics Studies (ARIES) project^35^. Blood samples from a sub-set of 1018 ALSPAC participants were taken at three time points for the child (neonatal, childhood (mean age 7.5 years) and adolescence (mean age 17.1 years)) and at two time points for the mothers (antenatal and during their child’s adolescence (mean age 17.1 years)). The DNA methylation data used in this study were from samples collected at the antenatal time point from mothers.

Normalisation was carried out using the *meffil* R package^55^. As part of default *meffil* functional normalisation, quality control checks were conducted to check sex, the median intensity methylated vs unmethylated signal for all control probes, dye bias, detection* p*-value, and presence of low bead numbers. Samples showing evidence of population stratification based on ALSPAC genetic data were removed (n = 29). Methylation status was quantified as the ratio of the methylated probe intensity and the overall intensity (sum of methylated and unmethylated probe intensities) resulting in a beta-value between 0 (completely unmethylated) and 1 (completely methylated)^56^. This beta-value represents the proportion of methylated cells within the sample. The impact of outliers was reduced by setting all methylation data points outside 3 times the interquartile range from the 25th to the 75th percentiles as missing. Final EWAS analyses included a total of *N* = 482,855 probes and 933 individuals.

#### Covariates

The following variables were included in the analyses to adjust for confounding effects^57–60^. Maternal age at delivery was derived from a questionnaire administered 8 weeks after the child’s birth using the self-reported date of birth of the mother and the date of birth of the child, measured as a continuous variable. Parity was categorised as either nulliparous or multiparous derived from a questionnaire administered at 18 weeks’ gestation. Maternal smoking during pregnancy was categorised as either never smoking during pregnancy or smoking during pregnancy, derived from a questionnaire administered at 23 weeks’ gestation. The following variables were derived from a questionnaire administered at 32 weeks’ gestation: whether the mother intended to breastfeed in the first month (recategorized as ‘yes’, ‘maybe’ or ‘no’ following methodology of Jones et al.^61^); maternal education, categorised as having completed qualifications below A-Levels or completed the qualifications of A-Levels and above; and occupational social class, categorised according to whether the mother or father had a manual or non-manual occupation (whichever was highest) according to the National Statistics Socio-economic Classification. For the methylation analyses, estimated cell counts were derived using the Houseman method^62^ and included as model covariates. Given the low ethnic diversity in our sample (< 3% identified as non-white), we did not include maternal ethnicity as a covariate.

### Statistical analysis

Analyses were carried out in R (version 4.1.0^63^). Descriptive statistics were calculated for the ALSPAC sample and for the ARIES subset. Any mothers with missing data for any of the exposure or covariates were removed, leaving only complete cases for the primary analyses. The final samples contained only singleton births. Descriptive statistics for each subset (both for the ALSPAC phenotype dataset and ARIES subset EWAS sample) were subsequently calculated, to check for similarity of samples across analyses.

#### Association of pre-pregnancy BMI with breastfeeding

We investigated whether pre-pregnancy BMI was associated with breastfeeding outcome (i.e., initiation and duration) using multiple regression. For each analysis, we first ran a univariate model considering only the association between pre-pregnancy BMI and the breastfeeding measure of interest. We subsequently repeated the analysis including covariates known previously to be associated with breastfeeding (intention to breastfeed, maternal smoking, age, occupational social class, parity, and education). For analysis of breastfeeding initiation, we used a logistic regression with binomial error structure; for analysis of continuous breastfeeding duration, we used a linear model with Gaussian error; and for analysis of categorical breastfeeding duration, we used ordinal logistic regression. We also conducted analyses on continuous breastfeeding duration as a time-to-event analysis using proportional hazards models, using the function survfit for univariate analyses and coxph for multivariate, from the *survival* package in R^64^.

#### Epigenome-wide association study (EWAS)

Three EWASs were carried out using multiple linear regression to estimate the association of DNA methylation in maternal antenatal peripheral blood with pre-pregnancy BMI (*N* = 724), breastfeeding initiation (*N* = 718), and breastfeeding duration as a continuous measure *(N* = 602*)*. For the EWAS of pre-pregnancy BMI, we modelled DNA methylation as the outcome. For EWASs of breastfeeding, we modelled breastfeeding as the outcome since we are interested in the effect of DNA methylation as a potential mediator on breastfeeding outcome. For analysis of breastfeeding initiation, we used a logistic regression with binomial error structure; for analyses of continuous breastfeeding duration and pre-pregnancy BMI, we used a linear model with Gaussian error. All models were adjusted for maternal age, parity, maternal smoking behaviour, maternal education, occupational social class, intention to breastfeed, cell-type estimates and 10 Surrogate Variables generated using Surrogate Variables Analysis to remove unmeasured confounding^65^. For the EWASs on breastfeeding, pre-pregnancy BMI was also included as a covariate, to account for potential direct effects of BMI on breastfeeding (in addition to those mediated through methylation). We conducted sensitivity analyses, exploring pre-pregnancy BMI as a categorical covariate, or without adjustment for BMI to assess direct effects of BMI on breastfeeding.

To control for multiple testing, we set the genome-wide threshold at *p* < 2.4 × 10^–7^^66^ as well as reporting results at a relaxed threshold of *p* < 1.0 × 10^–5^^67^. For each EWAS, the genomic inflation factor (lambda λ) was calculated to quantify the extent of the inflation and the excess false positive rate; quantile–quantile (Q–Q) plots were used as a visual tool to mark deviations of the observed distributions from the expected distribution of *p-*values. EWASs were carried out using R package *ewaff* (version: 0.0.2; https://github.com/perishky/ewaff).

We also ran a differentially methylated region (DMR) analysis using R package *dmrff*. DMRs were retained if they contained > 1 CpG and a *dmrff* adjusted *p*-value of < 0.05.

#### Candidate-CpG analysis

To further investigate the potential association between pre-pregnancy BMI and DNA methylation and whether methylation is subsequently associated with breastfeeding practice, a candidate gene analysis was carried out using a recent meta-EWAS of adult BMI across 18 studies by Do et al*.*^31^. In this analysis, we restricted ALSPAC EWAS results to consider only CpG sites that were associated with BMI in the Do et al*.* study, which identified 52 CpG sites passing a false discovery rate adjustment in their meta-EWAS of adult BMI. We then investigated whether these specific CpG sites were associated with pre-pregnancy BMI and breastfeeding practice in ALSPAC. We used a binomial test to assess agreement in direction of effect estimates between the BMI meta-EWAS of Do et al*.* and the EWAS of BMI in ALSPAC.

#### Mediation analysis

We used the Sobel test to assess if the relationship between pre-pregnancy BMI and breastfeeding (initiation or duration) was mediated by methylation. We tested all CpGs identified from all EWASs (i.e., on pre-pregnancy BMI, breastfeeding initiation and duration) at *p* < 1.0 × 10^–5^. We implemented this analysis using the R package *bda,* specifying pre-pregnancy BMI as the dependent variable, breastfeeding as the independent variable and methylation as the mediating variable.

### Supplementary Information

Supplementary Figure 1.

Supplementary Tables.

Supplementary Tables.

## Data Availability

The ALSPAC study website contains details of all the data that is available through a fully searchable data dictionary and variable search tool http://www.bristol.ac.uk/alspac/researchers/our-data/. ALSPAC data is available on request by application to the ALSPAC executive committee (ALSPAC-exec@bristol.ac.uk). The ALSPAC data management plan (available here: www.bristol.ac.uk/alspac/researchers/access/) describes in detail the policy regarding data sharing, which is through a system of managed open access.

## References

[1] Horta, B. L. & Victora, C. G. Long-term effects of breastfeeding. A systematic review. (World Health Organisation, 2013).

[2] Victora CG (2016). Breastfeeding in the 21st century: Epidemiology, mechanisms, and lifelong effect. The Lancet.

[3] World Health, O. & others. *Global strategy for infant and young child feeding*. (World Health Organization, 2003).

[4] McAndrew, F. *et al.* Infant feeding survey 2010. *Leeds: health and social care information Centre* **2** (2012).

[5] Who. WHO Factsheet; Obesity and overweight (2021).

[6] Amir LH, Donath S (2007). A systematic review of maternal obesity and breastfeeding intention, initiation and duration. BMC Pregn. Childbirth.

[7] Li R, Jewell S, Grummer-Strawn L (2003). Maternal obesity and breast-feeding practices. Am. J. Clin. Nutr..

[8] Flores TR, Mielke GI, Wendt A, Nunes BP, Bertoldi AD (2018). Prepregnancy weight excess and cessation of exclusive breastfeeding: A systematic review and meta-analysis. Eur. J. Clin. Nutr..

[9] Marshall NE, Lau B, Purnell JQ, Thornburg KL (2019). Impact of maternal obesity and breastfeeding intention on lactation intensity and duration. Matern. Child Nutr..

[10] Lovelady CA (2005). Is maternal obesity a cause of poor lactation performance?. Nutr. Rev..

[11] Turcksin R, Bel S, Galjaard S, Devlieger R (2014). Maternal obesity and breastfeeding intention, initiation, intensity and duration: A systematic review. Matern. Child Nutr..

[12] Lyons S, Currie S, Peters S, Lavender T, Smith DM (2018). The association between psychological factors and breastfeeding behaviour in women with a body mass index (BMI) ≥30 kg m^−2^: A systematic review. Obes. Rev..

[13] Nommsen-Rivers LA, Chantry CJ, Peerson JM, Cohen RJ, Dewey KG (2010). Delayed onset of lactogenesis among first-time mothers is related to maternal obesity and factors associated with ineffective breastfeeding. Am. J. Clin. Nutr..

[14] O'Sullivan EJ, Perrine CG, Rasmussen KM (2015). Early breastfeeding problems mediate the negative association between maternal obesity and exclusive breastfeeding at 1 and 2 months postpartum. J. Nutr..

[15] Hauff LE, Leonard SA, Rasmussen KM (2014). Associations of maternal obesity and psychosocial factors with breastfeeding intention, initiation, and duration. Am. J. Clin. Nutr..

[16] Keyes M (2023). Mediators of the association between maternal body mass index and breastfeeding duration in 3 international cohorts. Am. J. Clin. Nutr..

[17] Martin H, Thevenet-Morrison K, Dozier A (2020). Maternal pre-pregnancy body mass index, gestational weight gain and breastfeeding outcomes: A cross-sectional analysis. BMC Pregn. Childbirth.

[18] Jones PA, Takai D (2001). The role of DNA methylation in mammalian epigenetics. Science.

[19] Duncan EJ, Gluckman PD, Dearden PK (2014). Epigenetics, plasticity, and evolution: How do we link epigenetic change to phenotype?. J. Exp. Zool. Part B Mol. Dev. Evol..

[20] Dick KJ (2014). DNA methylation and body-mass index: A genome-wide analysis. The Lancet.

[21] Aslibekyan S (2015). Epigenome-wide study identifies novel methylation loci associated with body mass index and waist circumference. Obesity.

[22] Demerath EW (2015). Epigenome-wide association study (EWAS) of BMI, BMI change and waist circumference in African American adults identifies multiple replicated loci. Hum. Mol. Genet..

[23] Mendelson MM (2017). Association of body mass index with DNA methylation and gene expression in blood cells and relations to cardiometabolic disease: A mendelian randomization approach. PLOS Med..

[24] Sayols-Baixeras S (2017). DNA methylation and obesity traits: An epigenome-wide association study The REGICOR study. Epigenetics.

[25] Wahl S (2017). Epigenome-wide association study of body mass index, and the adverse outcomes of adiposity. Nature.

[26] Wilson LE, Harlid S, Xu Z, Sandler DP, Taylor JA (2017). An epigenome-wide study of body mass index and DNA methylation in blood using participants from the Sister Study cohort. Int. J. Obes..

[27] Geurts YM (2018). Novel associations between blood DNA methylation and body mass index in middle-aged and older adults. Int. J. Obes..

[28] Sun D (2019). Body mass index drives changes in DNA methylation. Circ. Res..

[29] Chen Y (2021). Impact of BMI and waist circumference on epigenome-wide DNA methylation and identification of epigenetic biomarkers in blood: An EWAS in multi-ethnic Asian individuals. Clin. Epigenet..

[30] Do WL (2021). Examining the association between adiposity and DNA methylation: A systematic review and meta-analysis. Obes. Rev..

[31] Do WL (2023). Epigenome-wide meta-analysis of BMI in nine cohorts: Examining the utility of epigenetically predicted BMI. Am. J. Hum. Genet..

[32] Odintsova VV (2019). DNA methylation signatures of breastfeeding in buccal cells collected in mid-childhood. Nutrients.

[33] Hartwig FP (2020). Association between breastfeeding and DNA methylation over the life course: Findings from the Avon longitudinal study of parents and children (ALSPAC). Nutrients.

[34] Gruzieva O (2019). DNA methylation trajectories during pregnancy. Genet. Epigenet..

[35] Relton CL (2015). Data resource profile: Accessible resource for integrated epigenomic studies (ARIES). Int. J. Epidemiol..

[36] van Iterson, M., van Zwet, E. W., Consortium, B. & Heijmans, B. T (2017). Controlling bias and inflation in epigenome- and transcriptome-wide association studies using the empirical null distribution. Genome Biol..

[37] Adab P, Pallan M, Whincup PH (2018). Is BMI the best measure of obesity?. BMJ.

[38] GTEX. https://gtexportal.org/home/.

[39] Sollis E (2023). The NHGRI-EBI GWAS Catalog: Knowledgebase and deposition resource. Nucleic Acids Res..

[40] Mulder RH (2021). Epigenome-wide change and variation in DNA methylation in childhood: Trajectories from birth to late adolescence. Hum. Mol. Genet..

[41] Min JL (2021). Genomic and phenotypic insights from an atlas of genetic effects on DNA methylation. Nat. Genet..

[42] Kent WJ (2002). The human genome browser at UCSC. Genome Res..

[43] Weng J, Couture C, Girard S (2023). Innate and adaptive immune systems in physiological and pathological pregnancy. Biology (Basel).

[44] Forsum E, Lof M (2007). Energy metabolism during human pregnancy. Annu. Rev. Nutr..

[45] Lesseur C (2013). Tissue-specific Leptin promoter DNA methylation is associated with maternal and infant perinatal factors. Mol. Cell Endocrinol..

[46] Sharp GC (2017). Maternal BMI at the start of pregnancy and offspring epigenome-wide DNA methylation: Findings from the pregnancy and childhood epigenetics (PACE) consortium. Hum. Mol. Genet..

[47] Ghildayal N (2022). Early-pregnancy maternal body mass index is associated with common DNA methylation markers in cord blood and placenta: A paired-tissue epigenome-wide association study. Epigenetics.

[48] Kong SK, Lee DT (2004). Factors influencing decision to breastfeed. J. Adv. Nurs..

[49] Morris C, Schofield P, Hirst C (2020). Exploration of the factors influencing attitudes to breastfeeding in public. J. Hum. Lact..

[50] Chang YS, Glaria AA, Davie P, Beake S, Bick D (2020). Breastfeeding experiences and support for women who are overweight or obese: A mixed-methods systematic review. Matern Child Nutr..

[51] Michels KB (2013). Recommendations for the design and analysis of epigenome-wide association studies. Nat. Methods.

[52] Boyd A (2013). Cohort profile: The ‘Children of the 90s’—the index offspring of the Avon Longitudinal Study of Parents and Children. Int. J. Epidemiol..

[53] Fraser A (2013). Cohort profile: The Avon longitudinal study of parents and children: ALSPAC mothers cohort. Int. J. Epidemiol..

[54] Seidell JC, Flegal KM (1997). Assessing obesity: Classification and epidemiology. Br. Med. Bull..

[55] Min JL, Hemani G, Davey Smith G, Relton C, Suderman M (2018). Meffil: efficient normalization and analysis of very large DNA methylation datasets. Bioinformatics.

[56] Du P (2010). Comparison of Beta-value and M-value methods for quantifying methylation levels by microarray analysis. BMC Bioinf..

[57] Noble, S. & Team, T. A. S (2001). Maternal employment and the initiation of breastfeeding. Acta Paediatr..

[58] Donath, S., Amir, L. & Team, T. A. S (2003). Relationship between prenatal infant feeding intention and initiation and duration of breastfeeding: a cohort study. Acta Paediatr..

[59] Donath S, Amir L (2004). The relationship between maternal smoking and breastfeeding duration after adjustment for maternal infant feeding intention. Acta Paediatr..

[60] Brion M-JA (2011). What are the causal effects of breastfeeding on IQ, obesity and blood pressure? Evidence from comparing high-income with middle-income cohorts. Int. J. Epidemiol..

[61] Jones CL, Culpin I, Evans J, Pearson RM (2020). Relative effects of breastfeeding intention and practice on maternal responsiveness. Infant Mental Health J..

[62] Houseman EA (2012). DNA methylation arrays as surrogate measures of cell mixture distribution. BMC Bioinf..

[63] R: A Language and Environment for Statistical Computing (R Foundation for Statistical Computing, Vienna, Austria, 2022).

[64] Therneau, T. A Package for Survival Analysis in R. R package version 3.2–7. 2020. *URL *https://CRAN.R-project.org/package=survival (2020).

[65] Leek JT, Johnson WE, Parker HS, Jaffe AE, Storey JD (2012). The sva package for removing batch effects and other unwanted variation in high-throughput experiments. Bioinformatics.

[66] Saffari A (2018). Estimation of a significance threshold for epigenome-wide association studies. Genet. Epidemiol..

[67] Rakyan VK, Down TA, Balding DJ, Beck S (2011). Epigenome-wide association studies for common human diseases. Nat. Rev. Genet..

