# Supplementary material for: Negative association between higher maternal pre-pregnancy body mass index and breastfeeding outcomes is not mediated by DNA methylation
Source: Sci Rep. 2024 Jun 25;14:14675. doi: 10.1038/s41598-024-65605-0 (PMC11199553; doi:10.1038/s41598-024-65605-0)

Supplementary information for: “**Negative association between higher maternal pre-pregnancy body mass index and breastfeeding outcomes is not mediated by DNA methylation”**

**Figure S1.** Plot from univariate survival analysis of cessation of breastfeeding for mothers of different BMI categories.


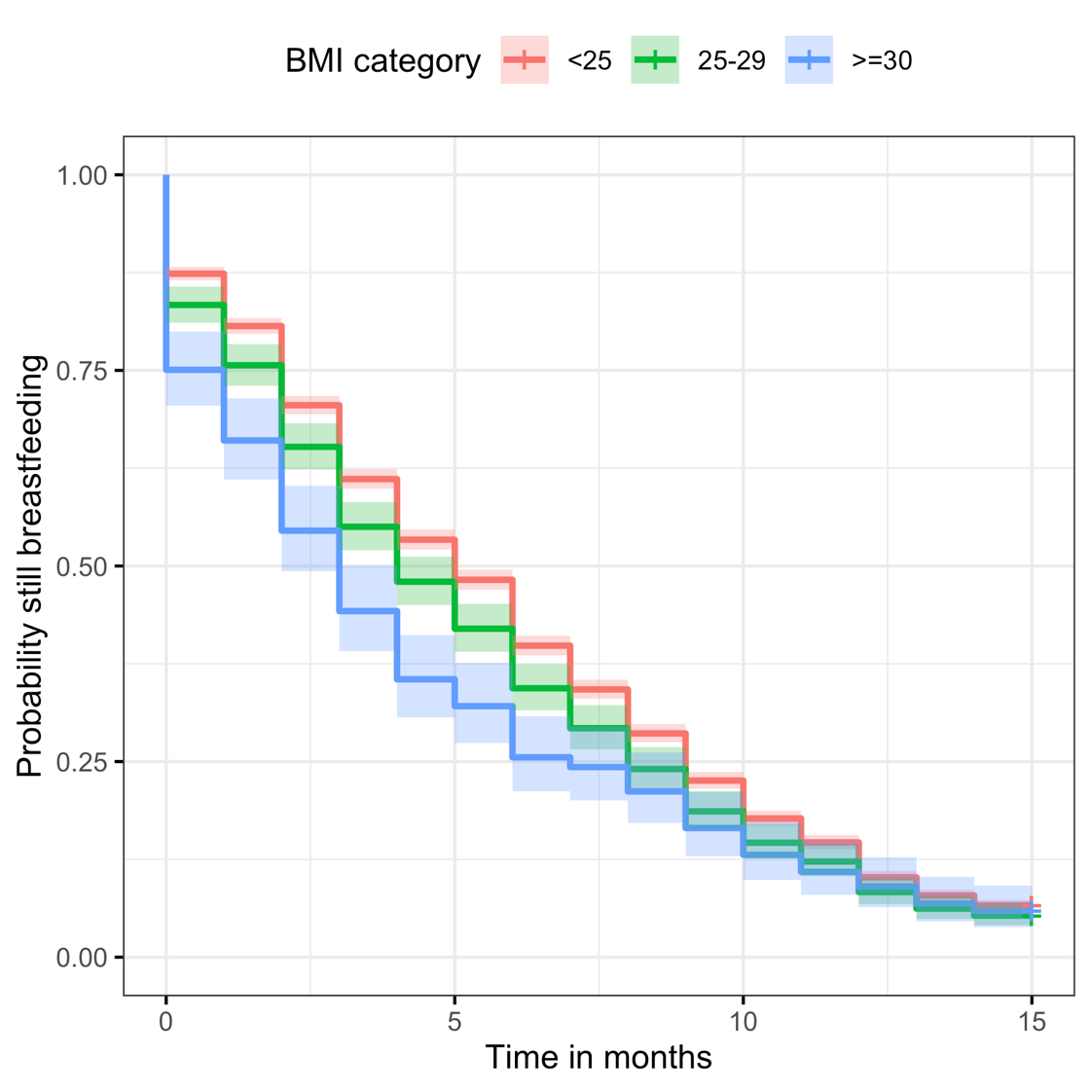

Supplement: Supplementary file 1 — Supplementary Figure 1. [file 41598_2024_65605_MOESM1_ESM.docx]
